# *w*Flu: Characterization and Evaluation of a Native *Wolbachia* from the Mosquito *Aedes fluviatilis* as a Potential Vector Control Agent

**DOI:** 10.1371/journal.pone.0059619

**Published:** 2013-03-26

**Authors:** Luke Anthony Baton, Etiene Casagrande Pacidônio, Daniela da Silva Gonçalves, Luciano Andrade Moreira

**Affiliations:** Laboratório de Malária, Centro de Pesquisas René Rachou -FIOCRUZ, Belo Horizonte, Minas Gerais, Brazil; Kansas State University, United States of America

## Abstract

There is currently considerable interest and practical progress in using the endosymbiotic bacteria *Wolbachia* as a vector control agent for human vector-borne diseases. Such vector control strategies may require the introduction of multiple, different *Wolbachia* strains into target vector populations, necessitating the identification and characterization of appropriate endosymbiont variants. Here, we report preliminary characterization of *w*Flu, a native *Wolbachia* from the neotropical mosquito *Aedes fluviatilis*, and evaluate its potential as a vector control agent by confirming its ability to cause cytoplasmic incompatibility, and measuring its effect on three parameters determining host fitness (survival, fecundity and fertility), as well as vector competence (susceptibility) for pathogen infection. Using an aposymbiotic strain of *Ae. fluviatilis* cured of its native *Wolbachia* by antibiotic treatment, we show that in its natural host *w*Flu causes incomplete, but high levels of, unidirectional cytoplasmic incompatibility, has high rates of maternal transmission, and no detectable fitness costs, indicating a high capacity to rapidly spread through host populations. However, *w*Flu does not inhibit, and even enhances, oocyst infection with the avian malaria parasite *Plasmodium gallinaceum*. The stage- and sex-specific density of *w*Flu was relatively low, and with limited tissue distribution, consistent with the lack of virulence and pathogen interference/symbiont-mediated protection observed. Unexpectedly, the density of *w*Flu was also shown to be specifically-reduced in the ovaries after bloodfeeding *Ae. fluviatilis*. Overall, our observations indicate that the *Wolbachia* strain *w*Flu has the potential to be used as a vector control agent, and suggests that appreciable mutualistic coevolution has occurred between this endosymbiont and its natural host. Future work will be needed to determine whether *w*Flu has virulent host effects and/or exhibits pathogen interference when artificially-transfected to the novel mosquito hosts that are the vectors of human pathogens.

## Introduction

There is currently both considerable interest and practical progress in using artificial infections of the endosymbiotic bacteria *Wolbachia* to reduce the capacity of wild mosquito populations to transmit human pathogens [1]–[3]. *Wolbachia* are obligate, intracellular, maternally-inherited, Gram-negative α-Proteobacteria, naturally-infecting a wide diversity of arthropods and crustaceans, which cause various forms of reproductive parasitism and other effects in their invertebrate hosts [4]–[6]. *Wolbachia* were first characterized [7], [8] and shown to cause “cytoplasmic incompatibility” in mosquitoes [9], [10]. Cytoplasmic incompatibility is a form of reproductive parasitism that increases the proportion of individuals in a host population infected with a given *Wolbachia* variant by suppressing the reproduction of those females that are either uninfected or infected with different *Wolbachia* variants. In the simplest scenario, uninfected females mated to *Wolbachia*-infected males do not produce viable offspring, while *Wolbachia*-infected females, whether they mate with uninfected males or those infected with the same *Wolbachia* variant, produce viable offspring themselves infected with *Wolbachia* [10]–[13].

Since its discovery, the phenomenon of cytoplasmic incompatibility has attracted attention as a possible means of controlling mosquito vector populations, either through direct reduction of vector population densities by mass release of incompatible males (population suppression, analogous to the sterile insect technique) [14]–[16] or as a mechanism to drive desirable traits associated with *Wolbachia* into vector populations (population replacement) [17]–[20]. In the last 15 years, interest in the use of *Wolbachia* as a vector control agent has intensified [2] with the development of techniques to artificially-transfect mosquitoes with *Wolbachia* [21]–[25], and the discovery that such infections can inhibit the development of vector-borne pathogens [26]–[33], decrease the survival of adult female mosquitoes [25], [30], [34], and reduce their vector biting rate [35], [36], thereby lowering the vectorial capacity of mosquito populations to transmit pathogens between human hosts [37]–[40]. Recent field trials have further demonstrated the proof-of-principle that release of relatively small seed populations of laboratory-reared mosquitoes artificially-infected with *Wolbachia* are sufficient to introduce and rapidly spread *Wolbachia* through wild uninfected mosquito populations [41], and a global effort is now being made to implement a *Wolbachia*-based dengue control strategy (http://eliminatedengue.com) [2].

Such vector control strategies require the identification of different *Wolbachia* strains with different characteristics appropriate for their intended application to vector control. For example, avirulent *Wolbachia* strains without fitness costs are most appropriate as gene drive mechanisms [20], while virulent endosymbiont strains reducing host survival are necessary to modulate the age-structure of vector populations [25], [37]–[40]. Different *Wolbachia* strains are also required for the multiple successive *Wolbachia* introductions that may be necessary to reverse or overcome the evolution of resistance in pathogens and/or vectors to *Wolbachia*-based approaches [19], [42], or to enable the application of *Wolbachia*-based strategies to vector mosquitoes already naturally-infected with *Wolbachia* [43], [44]. Additionally, *Wolbachia* strains may vary in their ability to infect novel hosts, such that identification of different strains may be required in order to successfully artificially-transfect mosquito vector species that are not naturally-infected with *Wolbachia* (e.g. *Anopheles*) [3], [18].

Recently, our laboratory colony of the neotropical mosquito *Aedes fluviatilis* (Lutz, 1904; = *Georgecraigius fluviatilis* [45]) was found to be infected with a novel strain of *Wolbachia*, which was named *w*Flu [27]. This mosquito has a cosmopolitan and widespread distribution throughout Central and South America, encompassing the region from southern Mexico in the north, through to northern Argentina in the south [46]. In general, *Ae. fluviatilis* is not regarded as a vector of human pathogens, although it can be both anthropophilic and peridomestic [46], and this mosquito has been shown experimentally to transmit Yellow Fever virus [47], historically being suspected as a vector of this virus in the field [48]. However, the ease of laboratory colonization and maintenance of *Ae. fluviatilis* [49], together with its high susceptibility to infection with the avian malaria parasite *Plasmodium gallinaceum* [50], means that this mosquito species is a particularly convenient and safe laboratory model for studying malaria and vector-parasite interactions [51]. Furthermore, as the transfer of *Wolbachia* between phylogenetically-similar hosts is thought to be easier than that between distantly-related hosts [24], [25], artificial infection of the mosquito species that are the vectors of human pathogens may be facilitated by using *Wolbachia* from other non-vector mosquito species [22].

Here we report preliminary characterization of *w*Flu in its native host *Ae. fluviatilis*, and evaluate its potential for use as a vector control agent by confirming its ability to cause cytoplasmic incompatibility, and measuring its effect on three parameters determining host fitness (survival, fecundity and fertility), as well as vector competence (susceptibility) for pathogen infection. Using an aposymbiotic strain of *Ae. fluviatilis* cured of its *Wolbachia* by antibiotic treatment, we show that *w*Flu causes incomplete, but high levels of, unidirectional cytoplasmic incompatibility, has high rates of transmission from mother to offspring, and no apparent fitness costs, indicating that this strain of *Wolbachia* has the capacity to effectively and rapidly disseminate through host populations. However, we also found, in contrast to previous studies, that *w*Flu did not reduce, and may even enhance, oocyst infection with *P. gallinaceum*. The stage-, sex- and tissue-specific density of *w*Flu was also determined, and related to the observed incomplete expression of CI, the lack of virulence of *w*Flu to its host, and the susceptibility to pathogen infection of *Ae. fluviatilis*. An unexpected observation not previously reported for mosquitoes and requiring further investigation was that *Wolbachia* densities in *Ae. fluviatilis* decrease within the ovaries during the process of oogenesis that occurs after bloodfeeding.

## Materials and Methods

### Ethics Statement

This study was carried out in strict accordance with the recommendations established by the Sociedade Brasileira de Ciência em Animais de Laboratório (SBCAL). The protocol for bloodfeeding mosquitoes on mice was approved by the Comissão de Ética no Uso de Animais (CEUA) Fiocruz (Licence Number LW-49/10), as were the protocols for malaria infection of chickens and their feeding to mosquitoes (Licence Numbers LW-18/12 and LW-38/12).

### Mosquitoes

The *Ae. fluviatilis* colony used was originally isolated in 1975 from the vicinity of FIOCRUZ Minas, Belo Horizonte, Brazil [49], [52]. The colony has since been continuously maintained at FIOCRUZ Minas, at 27±1°C, and 70±10% relative humidity, in a 12∶12 hour light:dark cycle. Larvae were reared in clean tap water and fed daily pelleted fish food (Goldfish Colour, Alcon, Camboriú, Santa Catarina, Cat. No. 0504-2). Adult mosquitoes were provided *ad libitum* with a 10% sucrose solution, and adult females were blood-fed on anaesthetized Swiss Webster mice for egg production.

### Generation of Ae. Fluviatilis Strain Cured of wFlu Infection

The wildtype (*wolb^+^*) colony of *Ae. fluviatilis* was cured of its native *Wolbachia* strain *w*Flu by mass treatment of adult females and males with the antibiotic tetracycline, as previously described [10], [53]. The adult mosquitoes were continually exposed *ad libitum* to a final concentration of 0.1 mg/ml of tetracycline hydrochloride (Sigma, St Louis, MO; Cat. No. T3383) in 10% sucrose solution, for approximately 10 to 14 days, in each of three consecutive generations. One thousand adults were treated in each generation, in order to minimize the effects of random genetic drift, and to maintain a colony size equivalent to that of wildtype (*wolb^+^*) *Ae. fluviatilis* colony. In each generation, individual females were randomly screened using conventional PCR to detect the presence of *Wolbachia* as described below. With the exception of the treatment with the antibiotic tetracycline, the wildtype (*wolb^+^*) and the antibiotic-treated (*wolb^−^*) strains of *Ae. fluviatilis* were otherwise maintained under the same standard insectary conditions described above. After withdrawal of the tetracycline from the antibiotic-treated (*wolb^−^*) strain of *Ae. fluviatilis*, experimental work was not initiated until two further generations, in order to allow re-acquisition of any environmental colony associated-microbiota, and recovery from any potential side-effects of the antibiotic treatment.

### Conventional PCR for Screening Wolbachia

Conventional PCR of the *Wolbachia surface protein* (*wsp*) gene [54] was used for routine screening of our *Ae. fluviatilis* colonies for the presence of *Wolbachia*. Crude DNA samples were prepared from individual mosquitoes by homogenization in 80 µl of “squash buffer” using a Mini-Beadbeater-16 (BioSpec, Bartlesville, Oklahoma; Cat. No. 607), as previously described [44]. Single or multiplex PCR reactions were performed on a Veriti® Thermal Cycler (Applied Biosystems, Carlsbad, CA) using previously published primers that amplify a 201 bp fragment of the *wsp* gene from *w*Flu (*WSPqPCR* forward: 5′ - ATC TTT TAT AGC TGG TGG TGG T - 3′; and *WSPqPCR* reverse: 5′ - GGA GTG ATA GGC ATA TCT TCA AT - 3′ [27]), and as a positive control to confirm DNA template quality primers that amplify a 266 bp fragment from the mosquito *actin-2* gene (forward: 5′ - GTC CGC GAT ATC AAG GAA AA - 3′; and reverse: 5′ - GTG TTG GCG TAC AGG TCC TT - 3′). The total reaction volume was 15 µl, consisting of a final concentration of 0.2 µM for each forward and reverse primer, 200 nM dNTPs, 20 mM Tris-HCL (pH 8.0), 50 mM KCl, 1.5 mM MgCl_2_, and 1 unit of *Taq* polymerase (Invitrogen, Grand Island, NY; Cat. No. 11615-010). The following three-step thermocycling conditions were used: an initial denaturation step at 95°C for 5 min; followed by 35 cycles of: 95°C for 30 sec, 55°C for 30 sec, and 72°C for 30 sec; and then a final extension step of 72°C for 5 min. PCR products were run on 2.0% agarose gels, along with a 100 bp DNA ladder (Promega, Madison, WI; Cat. No. G2101), and visualized using standard ethidium bromide staining.

### Experimental Crosses to Assess Fecundity and Determine the Occurrence of Cytoplasmic Incompatibility

Experimental crosses were performed between adults of the wildtype (*wolb^+^*) and the antibiotic-treated (*wolb^−^*) strains of *Ae. fluviatilis*. Pupae of each strain were sexed by examination of their terminalia, and each sex placed into separate cages to prevent uncontrolled mating after adult emergence (i.e., ensure virginity of the adults used in the experimental crosses). Two days after emergence from pupae, 25 virgin adults of each sex were mixed together in a single cage and allowed to mass-mate for two days. On the fifth day after emergence from pupae, the females in each cage were blood-fed on an anesthetized mouse for 30 min. Twenty-four hours after bloodfeeding, the females were removed from their cages and individually placed for oviposition into 50 ml Falcon tubes, lined at the bottom with filter paper and containing 5 ml of water. Five days later, the total number of eggs laid and the number of those eggs hatched were counted, individually for each female, using a stereomicroscope. The experiment was repeated twice, using two different generations of the laboratory colonies of the two mosquito strains. For those females with no hatched eggs, the spermathecae were checked for the presence of spermatozoa to confirm the occurrence of mating, and the *Wolbachia* infection status confirmed using the diagnostic PCR assay described above.

### Survival Analysis

Pupae of the wildtype (*wolb^+^*) and the antibiotic-treated (*wolb^−^*) strains of *Ae. fluviatilis* were sexed, and each sex placed into separate cages for emergence as adults. Virgin adults were collected on the day of emergence, and then placed into new cages, with 10 adults placed into each cage, separately for each sex, and for each strain of *Ae. fluviatilis*. The mosquitoes were provided *ad libitum* with a 10% sucrose solution throughout the duration of the experiment, and the mortality of the adults was recorded daily until all of the adults within each cage had died. The experiment was repeated twice, using two different generations of the laboratory colonies of the two mosquito strains.

### Infection of Ae. Fluviatilis with P. Gallinaceum

Five to 7-day-old adult females of both the wildtype (*wolb^+^*) and the antibiotic-treated (*wolb^−^*) strains of *Ae. fluviatilis* were fed serially for 30 minutes on the same gametocyte-positive chicks (5 to 30% parasitemia) infected with the 8A strain of *P. gallinaceum*, according to standard protocols [50], [55]. Non-blood-fed and/or not fully-engorged mosquitoes were removed within 24 hours, and the remaining fully-engorged mosquitoes were kept in standard insectary conditions until dissection 7 days after blood-feeding. Midguts were dissected in PBS, stained with a 2% solution of mercurochrome, and oocysts counted by light microscopy.

### Real-time Quantitative PCR (qPCR)

Real-time quantitative PCR was performed using the 7000 and 7500 Real-Time PCR Systems (Applied Biosystems). Crude DNA samples were extracted from whole individual mosquitoes, or pools of their dissected organs, as described above for conventional PCR, and diluted 1 in 10 with sterile DNase-free H_2_O. Relative quantitation of *Wolbachia* genome numbers was performed using the same primers given above for conventional PCR of the *wsp* gene (*WSPqPCR*), while the following previously published primers were used for amplification of a 80 bp fragment from the reference mosquito *actin-1* gene: forward: 5′ - ACC GAG CGT GGC TAC TCC TT - 3′; and reverse: 5′ - AGC GAC GTA GCA CAG CTT CTC - 3′ [27]. Absolute quantification of *Wolbachia* genome numbers was performed by construction of a standard curve using serial dilutions of the *w*Flu *wsp* sequence cloned into the pGEM®T-Easy plasmid (Promega, Madison, WI; Cat. No. A1360) [27]. A two-step reaction was performed with the following thermocycling conditions: an initial denaturation step at 95°C for 10 min, and then 35 cycles of: 95°C for 15 sec, followed by 60°C for 30 sec. The total reaction volume was 20 µl, consisting of 2X SYBR Green PCR Master Mix (Applied Biosystems, Carlsbad, CA; Cat. No. 4309155), a final concentration of 1 µM for each forward and reverse primer, and approximately 20 ng of sample DNA. Each sample was assayed in duplicate for both the *wsp* and *actin-1* genes. Separate gene-specific reaction efficiency corrections were empirically-determined using serial dilutions of a pool of all the samples assayed, while the same positive control sample was used on all plates, and used for inter-run calibration across plates. The raw C_t_ data were pre-processed, normalized and analysed qbasePLUS Premium, version 2.3 for Windows (Biogazelle NV, 2007–2012, Zwijnaarde, Belgium, http://www.biogazelle.com) [56].

### Statistical Analyses

All statistical analyses were performed using GraphPad Prism® version 5.01 for Windows (GraphPad Software, 1992–2007, San Diego, CA, www.graphpad.com).

## Results and Discussion

### wFlu is Native to Ae. Fluviatilis

*w*Flu has only previously been identified from our laboratory colony of *Ae. fluviatilis* [27], and it is theoretically possible that this *Wolbachia* strain was acquired sometime after colonization. However, sequencing of the *wsp* gene [54] and multilocus sequence typing (MLST) loci [57] confirms that our laboratory and field-collected *Ae. fluviatilis* are infected with the same strain of *Wolbachia* (manuscript in preparation), indicating that *w*Flu is native to *Ae. fluviatilis* and was presumably present in the founding individuals of our colony (i.e., *w*Flu was not acquired after isolation from the field and colonization in the laboratory).

### Tetracycline-treatment of Adults Cures Ae. Fluviatilis of wFlu Infection

In order to investigate the effect of *w*Flu upon its host, an aposymbiotic strain of *Ae. fluviatilis* without infection with its native *Wolbachia* (*wolb^−^*) was generated by mass tetracycline-treatment of wildtype adult mosquitoes infected with *w*Flu (*wolb^+^*) according to standard procedures [53]. The absence of *w*Flu in adult *Ae. fluviatilis* following antibiotic treatment was confirmed using a specific diagnostic PCR screen for the *Wolbachia* surface protein (*wsp*). From the second generation of antibiotic treatment onwards, *Wolbachia* was not detected in antibiotic-treated mosquitoes, but was always detected in wildtype individuals (data not shown). The *Ae. fluviatilis* colony appeared unaffected by antibiotic treatment, with no obvious reductions in fecundity, fertility or viability, either during tetracycline administration or in the period immediately following its withdrawal. This demonstrates that *w*Flu is a facultative (i.e., secondary) endosymbiont as observed for other mosquito-*Wolbachia* associations.

### wFlu causes Incomplete Unidirectional Cytoplasmic Incompatibility in Ae. Fluviatilis

**Figure 1:**
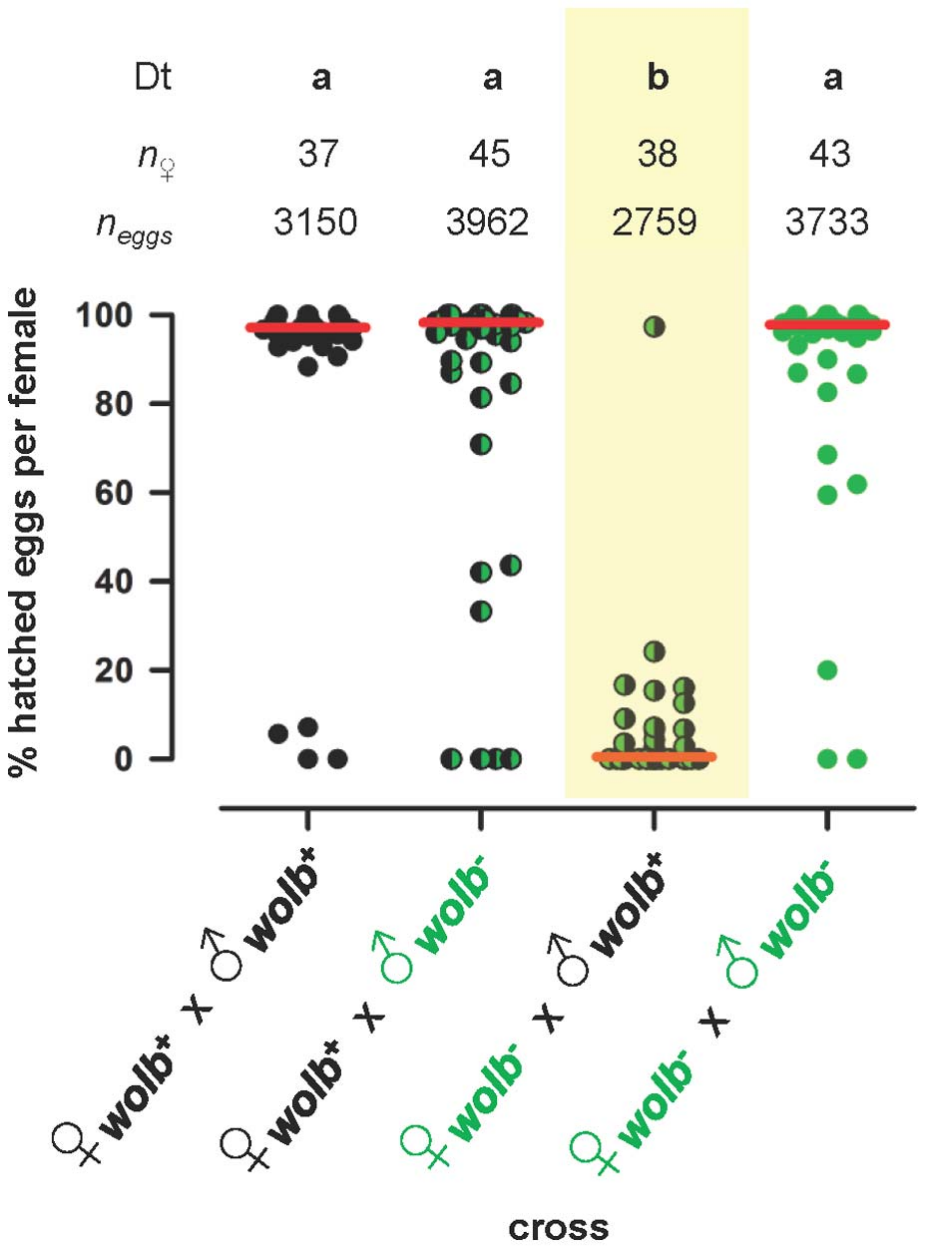
*w*Flu causes incomplete unidirectional cytoplasmic incompatibility in *Ae. fluviatilis*. Graph showing the percentage of eggs hatching in reciprocal crosses between the wildtype (*wolb^+^*) and antibiotic-treated (*wolb^−^*) strains of the mosquito *Ae. fluviatilis* (see *Materials and Methods* for details of the experimental design). Each circle represents a single adult female mosquito, while the red horizontal bars indicate the median number of hatched eggs per female. The data shown are pooled from two independent biological replicates (i.e., two different generations of the laboratory colony of *Ae. fluviatilis*). The total number of females (*n*_♀_) and the total number of eggs (*n*_eggs_) examined are indicated in the figure, above the data for each cross. The smallest group within either biological replicate comprised 16 females, which laid a total of 1109 eggs. All data from both biological replicates were analysed together using a Kruskal-Wallis test (*P*<0.0001), followed by pairwise comparison using Dunn’s test to determine which crosses differed significantly from one another. The letters (a, b) at the top of the figure, above the data for each cross, indicate the results of the Dunn’s test (Dt). Only the ♀*^wolb−^*×♂*^wolb+^* cross (highlighted in yellow) differed significantly from the other three crosses (b: in all three comparisons, *P*<0.001), which did not differ significantly from one another (a: in all three comparisons, *P*>0.05).

The successful application of *Wolbachia*-based vector control strategies requires endosymbionts that cause a high degree of cytoplasmic incompatibility [13]. In order to determine if *w*Flu causes cytoplasmic compatibility similar to that observed with other *Wolbachia* strains in different mosquito species [10], [58], [59], [60], reciprocal crosses were performed between the wildtype (*wolb^+^*) and the antibiotic-treated (*wolb^−^*) strains of *Ae. fluviatilis* (Figure 1). When uninfected females (♀*^wolb−^*) were crossed with *Wolbachia*-infected males (♂*^wolb+^*), a median of only 0.54% of eggs hatched per female, while a median of 97.2 to 98.5% of eggs hatched per female in the other three crosses. The median number of hatched eggs in the “incompatible” ♀*^wolb−^*×♂*^wolb+^* cross was significantly lower than that from the three “compatible” crosses, which did not differ significantly from one another (see legend to Figure 1 for results of the statistical analyses).

However, there was also appreciable variation in hatching rates between different females *within* each of the four experimental crosses, which is masked when only the median hatch rate per female is considered (Figure 1). In general, this variation was due to a minority of females exhibiting extreme phenotypes. Although only a median of 0.54% of eggs hatched per female in the “incompatible” ♀*^wolb−^*×♂*^wolb+^* cross, when the data from individual females were pooled and analysed *en masse*, overall 6.4% (176/2759) of eggs hatched, with 73 (41.5% of those hatching) coming from a single female. In the “incompatible” ♀*^wolb−^*×♂*^wolb+^* cross, no eggs hatched for 50% (19/38) of females, 1.1 to 24.2% of eggs hatched for 46.2% (18/39) of females, and 97.3% of eggs hatched for one female. In contrast, in the three “compatible” crosses, no eggs hatched for between 4.7 to 8.9% of females, some but not all eggs hatched for between 60.0 to 73.0% of females, while all eggs hatched for 21.6 to 31.1% of females. The proportions of females with no/some/all eggs hatched were significantly different in the “incompatible” ♀*^wolb−^*×♂*^wolb+^* cross compared to those in the three “compatible” crosses (χ^2^ = 44.65, d.f. = 2, *P*<0.0001), which did not differ significantly from one another (χ^2^ = 2.217, d.f. = 4, *P* = 0.6959).

Microscopic examination of the spermathecae confirmed the occurrence of successful mating in at least greater than 90% of those females with no hatched eggs (and all such females from the “incompatible” ♀*^wolb−^*×♂*^wolb+^* cross), with at least one of the three capsules comprising the spermathecae containing live spermatozoa (data not shown). Diagnostic PCR for the *wsp* gene was also used to confirm the appropriate *Wolbachia*-infection status of the crossed individuals of both sexes (data not shown). In particular, diagnostic PCR for *wsp* confirmed that (i) males from the incompatible cross were infected with *Wolbachia*, and that (ii) females with hatched eggs from the incompatible cross were uninfected with *Wolbachia*. As there was no evidence of contamination between the wildtype (*wolb^+^*) and the antibiotic-treated (*wolb^−^*) strains, we concluded that *w*Flu causes partial, incomplete cytoplasmic incompatibility in its natural host *Ae. fluviatilis*.

In other mosquito species, natural *Wolbachia* infections may cause either partial or complete cytoplasmic incompatibility [10], [59], [60], [61], [62], [63], [64], [65], although the latter is generally encountered, and apparently more so than in other Diptera [13]. The level of cytoplasmic incompatibility caused by *w*Flu in *Ae. fluviatilis* is high, but still appreciably lower, and more variable, than that reported in some other mosquito species that have been examined. The causes of partial cytoplasmic incompatibility and variation in its expression are not well understood, and may be determined by host, endosymbiont and environmental factors [60], [66], [67]. In mosquitoes and other insects, a threshold density of *Wolbachia* in the testes has been suggested to be required for efficient sperm modification, and hence the expression of cytoplasmic incompatibility [68]–[72]. In *Ae. fluviatilis* males, the density of *w*Flu is often low and highly variable (see real-time quantitative PCR data below and Supporting Information Figure S1), suggesting that some males may have insufficient numbers of *Wolbachia* for efficient sperm modification. Furthermore, we used 3 to 4 day-old males in our crossing experiments, but the density of *w*Flu does not appear to reach its peak in adults of this sex until at least 6 days post-emergence (see below and Supporting Information Figure S1). Another not mutually exclusive explanation for the lack of complete cytoplasmic incompatibility is that *w*Flu is polymorphic and consists not only of so-called “*mod^+^ resc^+^*” variants, capable both of inducing sperm *mod*ification in males and *resc*uing the fertilized eggs of females, but also “*mod^−^ resc^+^*” variants incapable of modifying sperm but capable of rescuing fertilized eggs (see [73] for a detailed explanation of the *mod resc* model). Accordingly, when a male infected with a *mod^−^ resc^+^* variant of *Wolbachia* mates with an uninfected female, cytoplasmic incompatibility does not occur [74], [75]. Further experiments using artificial selection [66] would be necessary to determine if the *w*Flu in our laboratory colony of *Ae. fluviatilis* consists of more than one *mod resc* variant.

### wFlu has No Effect on the Fitness of Ae. Fluviatilis

**Figure 2:**
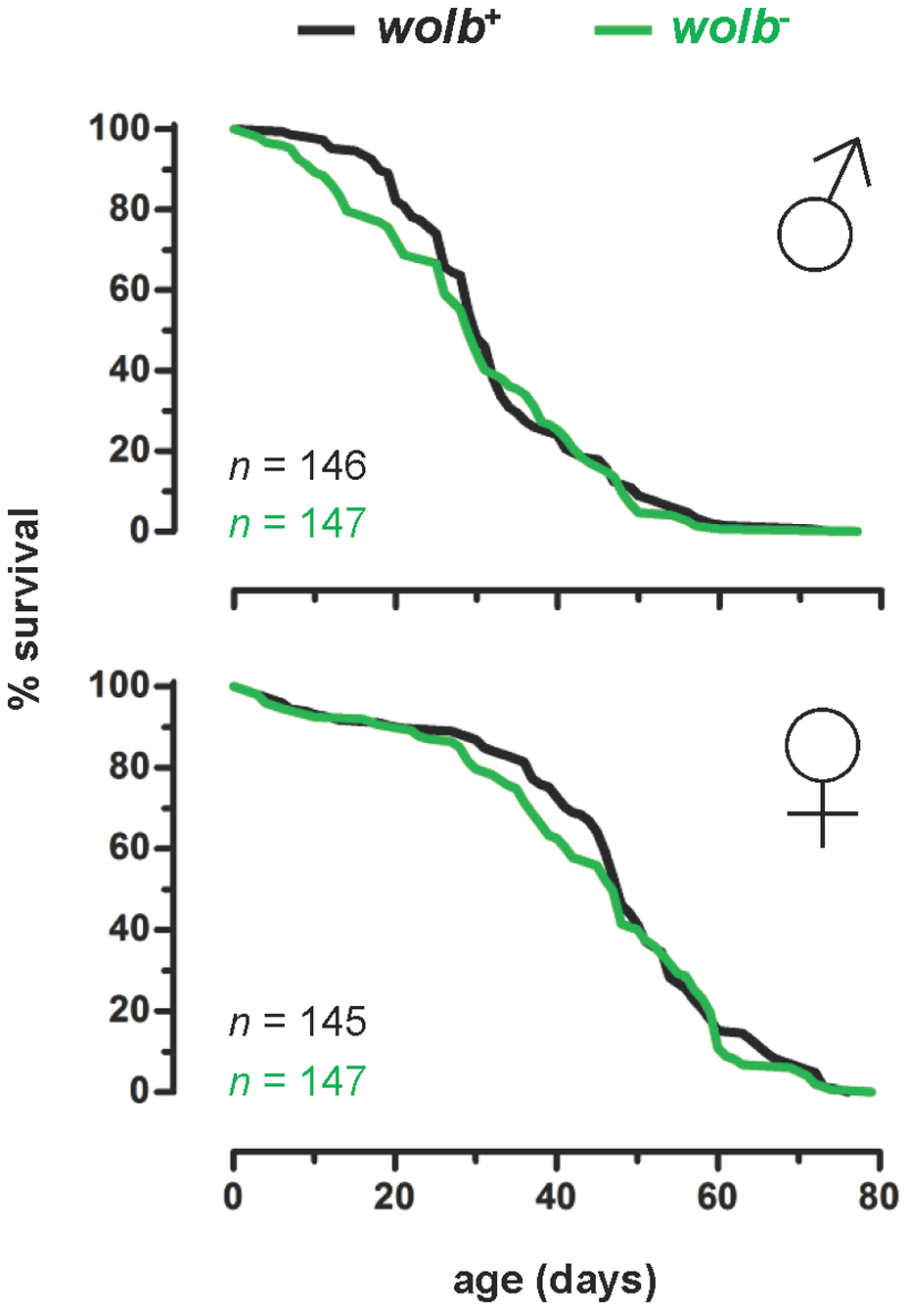
*w*Flu has no effect on the longevity of adult *Ae. fluviatilis*. Graphs showing the Kaplan-Meier survival curves for sugar-fed adult males (♂, top graph) and females (♀, bottom graph) of the wildtype (*wolb^+^*) and antibiotic-treated (*wolb^−^*) strains of the mosquito *Ae. fluviatilis*. The data shown were pooled from two independent biological replicates (i.e., two different generations of the laboratory colony of *Ae. fluviatilis*), and analysed together (see *Materials and Methods* for details of the experimental design). The survival curves for each sex did not differ significantly between wildtype (*wolb^+^*) and antibiotic-treated (*wolb^−^*) individuals (log-rank (Mantel-Cox) test: males, χ^2^ = 0.6743, *P* = 0.4116; and females, χ^2^ = 0.5850, *P* = 0.4444; and Mantel-Haenszel hazard ratios: males, ratio = 0.9046, 95% CI 0.7121 to 1.1490; and females, ratio = 0.9103, 95% CI 0.7154 to 1.1580).

**Figure 3:**
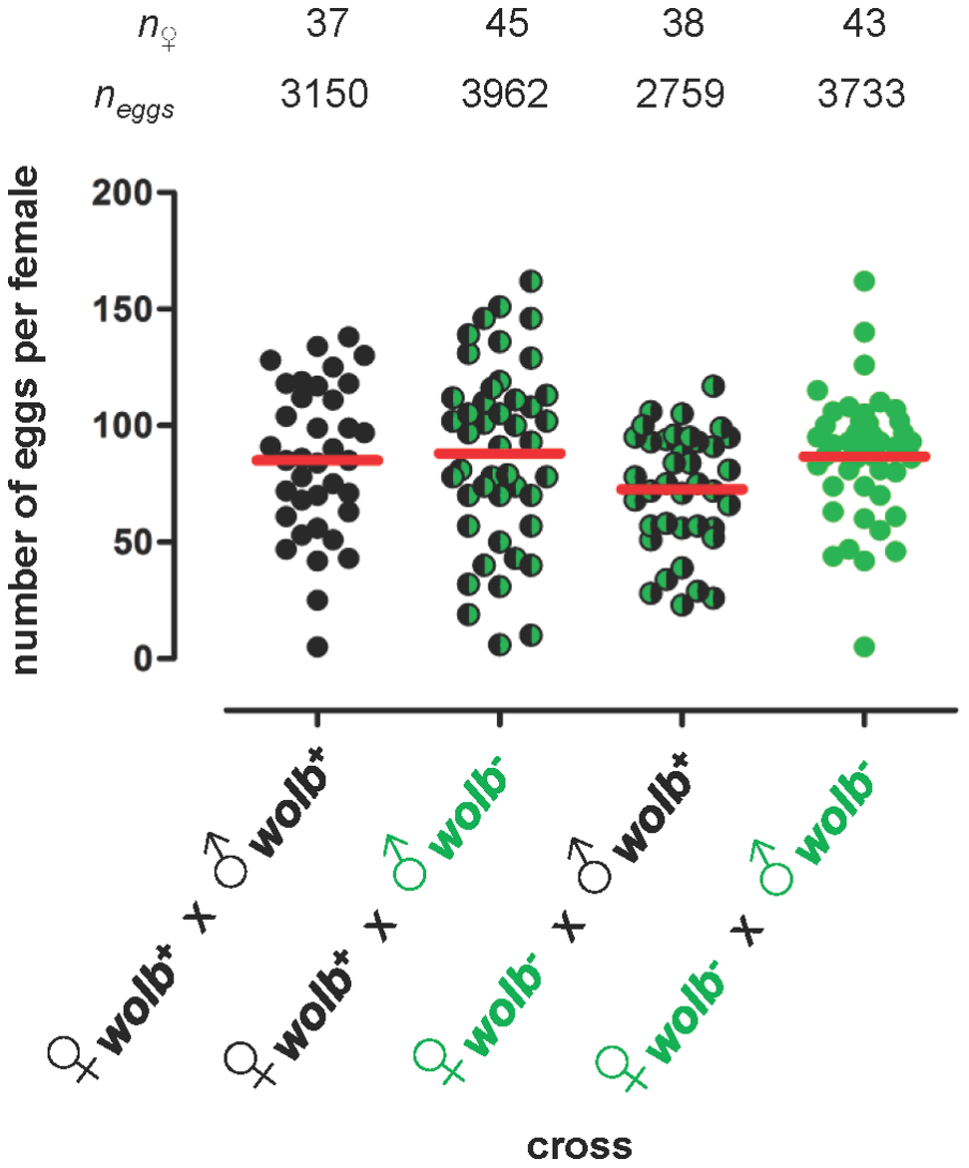
*w*Flu has no effect on the fecundity of female *Ae. fluviatilis*. Graph showing the number of eggs laid in reciprocal crosses between the wildtype (*wolb^+^*) and antibiotic-treated (*wolb^−^*) strains of the mosquito *Ae. fluviatilis*. Each circle represents a single adult female mosquito, while the red horizontal bars indicate the mean number of eggs per female. The data shown are from the same two experiments presented in Figure 1. The total number of eggs laid per female did not differ significantly between the four reciprocal crosses (ANOVA, *F*_(3, 159)_ = 2.008, *P* = 0.115).

Previous studies in mosquitoes have shown that native *Wolbachia* have variable effects on the fitness of their natural hosts: decreasing, increasing or not affecting host survival and/or reproduction [43], [61]–[65], [76]–[80]. Such host fitness effects are important for *Wolbachia*-based vector control strategies because they can: (i) affect the ability of *Wolbachia* to invade and spread through host populations [11]–[13], [62], [64], [81], and (ii) alter the age-structure of host populations, thereby modulating their capacity to transmit vector-borne pathogens [25], [37]–[40], [80]. Accordingly, three parameters that determine host fitness – survival, fecundity and fertility – were measured for wildtype (*wolb^+^*) and the antibiotic-treated (*wolb^−^*) strains of *Ae. fluviatilis* (Figures 2 and 3, and Figure 1).

Comparison of the daily survival rates of sugar-fed adults showed that *w*Flu had no effect on the longevity of either male or female *Ae. fluviatilis*, although, as expected for mosquitoes, the survival of males and females, independent of the presence or absence of *Wolbachia* infection, were significantly different from one another (data not shown) (Figure 2). Anecdotal observations also suggest that the survival of adult females of the wildtype (*wolb^+^*) and the antibiotic-treated (*wolb^−^*) strains of *Ae. fluviatilis*, in the week after feeding on either uninfected or *P. gallinaceum*-infected blood (see below), is not appreciably different from either sugar-fed individuals or one another (in all three instances, daily survival is >95%) (data not shown).

*w*Flu also had no effect on the fecundity (potential reproduction) of *Ae. fluviatilis* (Figure 3). Although the observed average total number of eggs laid per female was lower in the “incompatible” ♀*^wolb−^*×♂*^wolb+^* cross, overall there were no significant differences between the four experimental crosses in the total number of eggs laid per female (Figure 3). Additionally, the data presented in Figure 1 demonstrate that the number of larvae hatched per female did not differ significantly between the three compatible crosses (ANOVA, *F*_(2, 122)_ = , *P* = 0.816), indicating that *w*Flu also has no effect on the fertility (realized reproduction) of the wildtype (*wolb^+^*) and the antibiotic-treated (*wolb^−^*) strains of *Ae. fluviatilis*.

**Figure 4:**
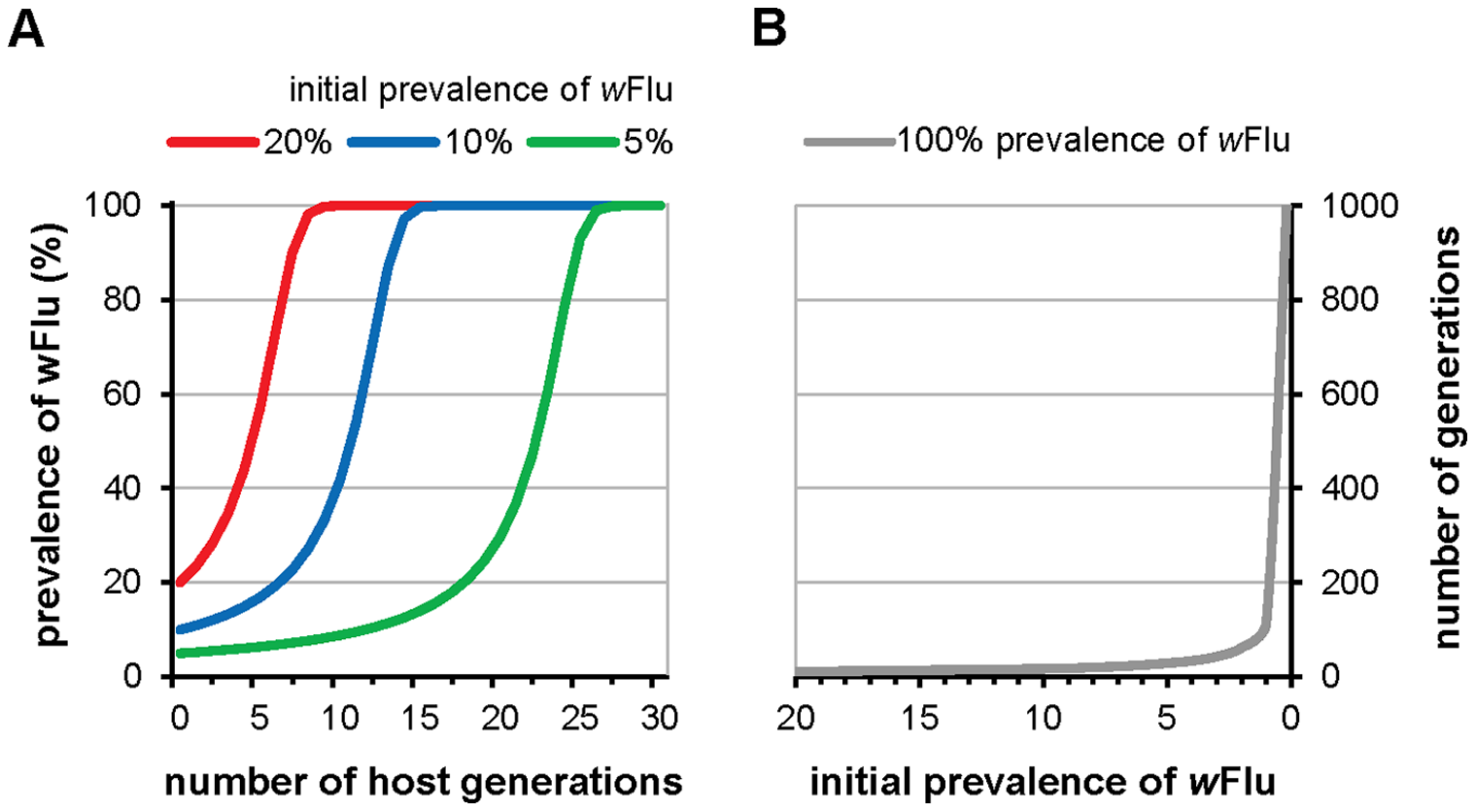
Mathematical modelling of the ability of *w*Flu to invade host populations. Theoretical prediction of the ability of the *Wolbachia* strain *w*Flu to invade uninfected host populations using the empirically-determined laboratory-based parameter estimates observed in this study for *w*Flu in its native host *Ae. fluviatilis*, and equation (1) from Dobson et al [62], modified from Turelli & Hoffmann [130]. Graph A shows three different predictions of the rate of spread of *w*Flu based upon three different initial prevalences of *w*Flu in the host population (5, 10 and 20%), which can be interpreted as the size of released *Wolbachia*-infected seed populations relative to the uninfected host population during a vector control programme. Graph B shows the general relationship between the initial prevalence of *w*Flu and the number of host generations required for *w*Flu to attain 100% prevalence in the host population. Coloured circles indicate values for the initial prevalences used in Graph A. The following parameter values were used to calculate the prevalence of infection (*p*) at generation time (*t*) by iteration: μ, the maternal transmission efficiency (the proportion of uninfected offspring produced by infected mothers) = 0.0 (i.e., complete maternal transmission was assumed; see main text for justification); *H*, the relative egg hatching rate (the ratio of hatched eggs from infected versus uninfected mothers) = 0.071; α, the relative fitness of infected versus uninfected females = 1.0 (i.e., no difference in fitness was inferred based on the survival and fecundity data presented in Figures 2 and 3, respectively). *H* was calculated using pooled total egg counts for the compatible and incompatible crosses shown in Figure 1, rather than the average hatch rate per female, in order to provide a more conservative estimate of the strength of cytoplasmic incompatibility (i.e., to account for the variation in the expression of cytoplasmic incompatibility observed with *w*Flu – see main text for detailed explanation).

Overall, our observations indicate that under laboratory conditions *w*Flu has no effect on the fitness of *Ae. fluviatilis*, as might be theoretically-expected for a vertically-transmitted endosymbiont in association with its coevolved natural host [12], [73], [82]–[84]. *w*Flu, therefore, appears to be avirulent (i.e., it has no fitness cost), and this characteristic implies that *w*Flu has a high capacity to invade host populations (see below and Figure 4). However, the avirulence of *w*Flu also implies that this strain of *Wolbachia* probably cannot be used to modify the age-structure of vector populations [25], [37]–[40], as life-shortening virulence appears to be a *Wolbachia* strain-specific property, rather than determined by host background [25], [30], [31], [34], [85] (i.e., when artificially-transfected to a new host, *w*Flu is likely to remain avirulent and not significantly affect host survival). Although the apparent absence of fitness costs, together with its associated high levels of cytoplasmic incompatibility, suggest that *w*Flu may be appropriate for use in population suppression strategies involving the release of incompatible males artificially-infected with this *Wolbachia* strain [15], [16], [86], [87].

### wFlu is Predicted to Rapidly Invade Host Populations

The ability of *Wolbachia* to invade and spread through host populations has been mathematically-modelled, and is known to depend on several parameters, including: (i) the level of cytoplasmic incompatibly, (ii) the maternal transmission efficiency (i.e., the proportion of offspring who fail to inherit the infection from their infected mother), and (iii) any host fitness costs associated with *Wolbachia* infection [11]–[13], [62], [64], [81], [88], [89]. We used the empirical data on the survival, fecundity and fertility of laboratory-reared *Ae. fluviatilis* described above to model the ability of *w*Flu to invade host populations using equation (1) from Dobson et al [62] (Figure 4). We have not formally measured the maternal transmission efficiency of *w*Flu, but routine random screening of our wildtype *Ae. fluviatilis* (*wolb^+^*) colony over a period of more than one year (unpublished observations) and the 204 individuals used for real-time quantitative PCR (see below) has failed to detect uninfected mosquitoes. The maternal transmission efficiency of *w*Flu was, therefore, taken to approximate 100% for the purposes of the model presented in Figure 4, and is consistent with estimates from other native *Wolbachia* strains infecting mosquitoes [13], [63], [65], [90]. Although the parameters determined for a specific host under laboratory conditions do not necessarily translate to those for different hosts and/or the field [13], [60], [67], [91], and should be interpreted with caution when extrapolated to new scenarios, mathematical modelling does indicate that *w*Flu has the capacity to rapidly invade host populations, using the relatively small seed populations envisioned for vector control programmes (Figure 4A). As *w*Flu has an approximately 100% maternal transmission efficiency and no known associated fitness costs, theoretically, at least under the conditions modelled, *w*Flu also does not require a threshold prevalence in order to spread through the host population, but will inexorably invade the latter regardless of its initial prevalence [11], [12]. However, it should be noted that the number of host generations required to attain 100% prevalence increases exponentially as the initial prevalence of *w*Flu falls below approximately 1% (Figure 4B). The apparent lack of virulence and fitness costs associated with *w*Flu, together with its high capacity for population invasion, suggest that in these respects this *Wolbachia* strain would make an excellent gene drive mechanism [17]–[20], if it retains similar characteristics when artificially-transferred to the novel hosts that are vectors of human pathogens. The results of the mathematical model also imply that *w*Flu is likely to be widespread in wild populations, throughout the geographical range, of its natural host *Ae. fluviatilis*.

### wFlu does not Inhibit Plasmodium Infection in Ae. Fluviatilis

**Figure 5:**
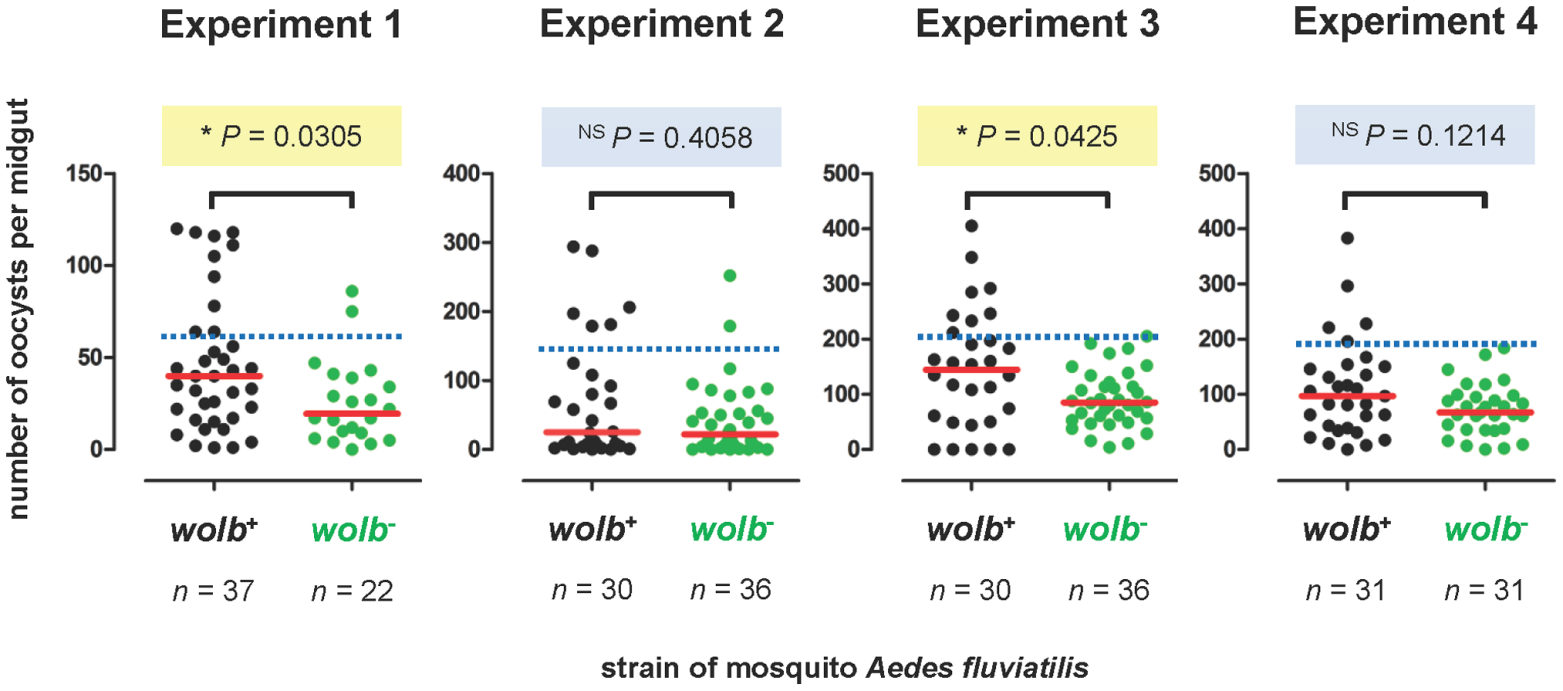
*w*Flu does not inhibit *Plasmodium* in *Ae. fluviatilis*. Graphs showing the number of oocyst stage malaria parasites observed on the midguts of wildtype (*wolb^+^*) and antibiotic-treated (*wolb^−^*) strains of the mosquito *Ae. fluviatilis* 7 days after infection with the avian malaria parasite *P. gallinaceum*. Each circle represents a single midgut from an adult female mosquito, while the red horizontal bars indicate the median number of oocysts per midgut. The data shown are from four independent biological replicates (i.e., four different generations, after antibiotic treatment, of the laboratory colony of *Ae. fluviatilis*). The numbers of oocysts per midgut were compared separately for each biological replicate (i.e., generation) using a Mann-Whitney *U* test. * = significantly different; NS = not significantly different. The dashed blue lines indicate the threshold used in Figure 6 to classify mosquitoes as having either relatively low or high *P. gallinaceum* oocyst infections.

**Figure 6:**
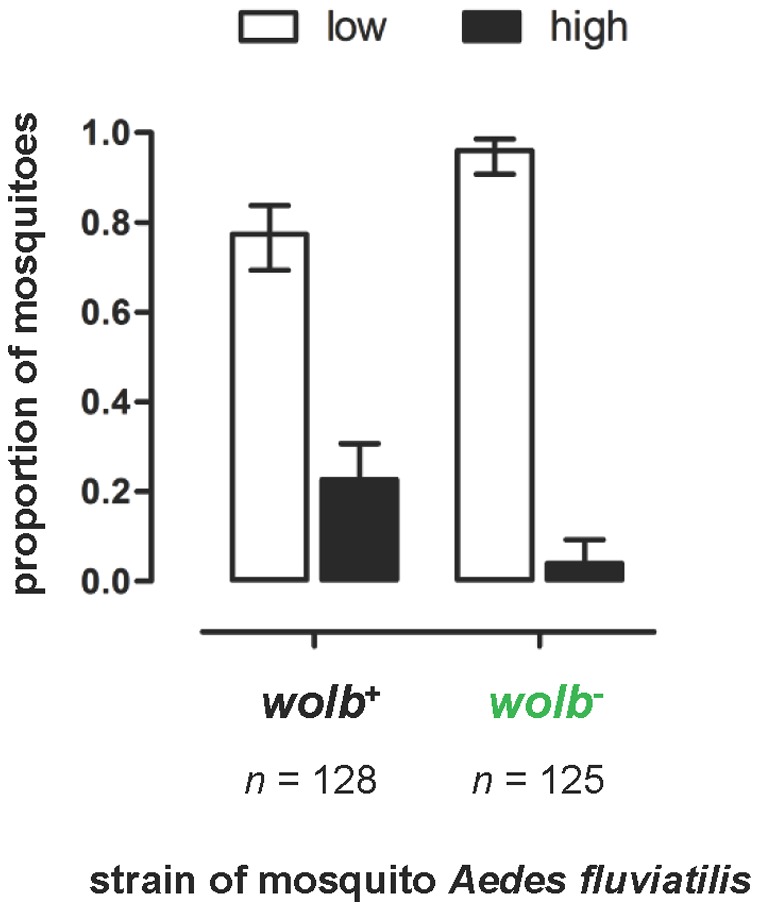
*w*Flu increases the intensity of *Plasmodium* oocyst infection in a subset of *Ae. fluviatilis*. Graph showing the proportion of wildtype (*wolb^+^*) and antibiotic-treated (*wolb^−^*) *Ae. fluviatilis* mosquitoes with low and high levels of *P. gallinaceum* oocyst infection. Data shown are the same as those presented in Figure 5, but classified as a dichotomous trait. Individual mosquitoes were classified as having either relatively low or high levels of oocyst infection according to whether their number of oocysts was smaller or greater than half the highest number of oocysts observed for that experiment (this threshold is indicated by the dashed blue lines in Figure 5). The data so classified were pooled for all 4 experiments and analysed together using a 2×2 contingency table, which showed that proportion of mosquitoes with relatively low or high oocyst infections was significantly different between the wildtype (*wolb^+^*) and antibiotic-treated (*wolb^−^*) strains of *Ae. fluviatilis* (χ^2^ = 18.92, d.f. = 1, *P*<0.0001). Error bars indicate 95% confidence intervals of the proportions.

Previous studies with a variety of mosquito-borne pathogens, including various malaria parasite species, filarial nematodes, and arboviruses have shown that *Wolbachia* may either reduce [26]–[32], [92], increase [32], [33] or have no effect [80], [93]–[96] on the susceptibility of mosquitoes to pathogen infection. In order to determine whether the *w*Flu in its natural host might influence vector competence and inhibit the development of oocysts of malaria parasites, *P. gallinaceum* infection was compared between the wildtype (*wolb^+^*) and the antibiotic-treated (*wolb^−^*) strains of *Ae. fluviatilis* in 4 different generations after tetracycline-treatment of the colony (Figure 5). The mosquito *Aedes aegypti* artificially-infected with the virulent *Wolbachia* strain *w*MelPop from the fruit fly *Drosophila melanogaster* [25], [97] has previously been shown to have reduced levels of oocyst infection with *P. gallinaceum* [27]. In contrast, we found that *P. gallinaceum* oocyst infection in *Ae. fluviatilis* was not inhibited by the presence of the native *w*Flu, and was even increased (Figure 5). In two of the generations tested (Experiments 1 and 3), the intensity of oocyst infection was significantly higher in wildtype (*wolb^+^*) compared to the antibiotic-treated (*wolb^−^*) strains of *Ae. fluviatilis*. In the two other generations tested (Experiments 2 and 4), there were no significant differences in the intensity of oocyst infection between the wildtype (*wolb^+^*) and the antibiotic-treated (*wolb^−^*) strains of *Ae. fluviatilis*, although in both instances the observed median level of oocyst infection was marginally higher in the wildtype (*wolb^+^*) than the antibiotic-treated (*wolb^−^*) strain of *Ae. fluviatilis*. The cause of the variation in the effect of *w*Flu on oocyst infection between experiments is unclear; it does not show a relationship with the number of generations after antibiotic treatment (i.e., it is apparently not a consequence of host adjustment following removal of its native *w*Flu). Figure 5 suggests that in at least 3 of the 4 experiments, the presence of *w*Flu is associated with an expansion in the upper range of oocyst infection, rather than an elevation of the number of oocysts in each individual (i.e., the lower range of oocyst infection is similar in mosquitoes with and without *Wolbachia*). When mosquitoes were classified according to those with low and high levels of malaria infection, *w*Flu is associated with a significant increase in the proportion of individuals with heavy oocyst infections (Figure 6), suggesting that the enhancing effect of *w*Flu on malaria infection is specific to a subset of the individuals examined, which may account for the variability observed between different experiments.

In general, at least in mosquitoes, artificial *Wolbachia* infections in novel hosts seem to be more effective at inhibiting pathogen development, than *Wolbachia* in natural host-endosymbiont associations [2], [32], although there are exceptions [33], [92]. Natural *Wolbachia* infection has no effect on the level of malaria parasite infection in mosquito hosts [80], while artificial *Wolbachia* infections may reduce oocyst infection [27], [29], [30], [33], but can also have opposing effects on oocyst infection with different malaria parasite species in the same mosquito host (i.e., the inhibitory or enhancing effect of *Wolbachia* is parasite-specific) [30], [33]. The effect of *Wolbachia* on vector competence, therefore, is complex, and not necessarily a simple function of the naturalness or not of the host-endosymbiont association. Our observations on the effect of *w*Flu on *P. gallinaceum* infection in *Ae. fluviatilis* are consistent with the notion that native *Wolbachia* are less likely to inhibit pathogen development, but do not explain the occurrence of enhanced oocyst infection associated with *w*Flu. The cause of *Wolbachia*-mediated modulation of vector competence for pathogen infection has not yet been fully-determined, but activation of host immune responses and/or competition for host resources have both been proposed as mechanisms reducing pathogen infection in mosquito hosts artificially-infected with *Wolbachia* [2], [26], [27]. However, it is not apparent how either mechanism would account for the occurrence of increased pathogen densities that are sometimes associated with *Wolbachia* (our data presented here and [32], [33]). Artificial *Wolbachia*-infections in novel mosquito hosts stimulate potent immune responses [26]–[30], [32], [98]–[100], which are thought to be absent or much-reduced in natural host-endosymbiont associations [32], [101], [102]. The lack of pathogen interference observed with *w*Flu implies an absence of both immune activation and resource competition, consistent with the avirulence (see above), low density and limited tissue distribution of *w*Flu in its native host *Ae. fluviatilis* (see below), especially in comparison to that observed in *Ae. aegypti* artificially-infected with *w*MelPop, which inhibits *P. gallinaceum* [2], [27]. Alternative explanations for *Wolbachia*-mediated pathogen enhancement could be immune suppression/diversion or, at least in natural host-endosymbiont associations, that the artificial loss of *Wolbachia* creates a disturbance in normal host physiology, which is adapted to the presence of the endosymbiont [103], that inhibits pathogen development. Such a scenario would imply a certain degree of mutualism between *w*Flu and its native host, as has recently been suggested in another host-*Wolbachia* association [104], although, as might be expected according to this hypothesis, we have not observed any apparent detrimental phenotypic effect of removing *w*Flu from *Ae. fluviatilis*.

We should emphasize here that *Ae. fluviatilis* is not a natural vector of *P. gallinaceum* [50], and the absence of a protective effect of this *Wolbachia* strain against this malaria parasite species should not be interpreted as evidence against the general hypothesis of a selective evolutionary advantage for symbiont-mediated protection [105], [106]. However, our observations do indicate that symbiont-mediated protection is not a generalized systemic response active against any pathogen, and further that *w*Flu may enhance *Plasmodium* infection demonstrates that *Wolbachia* may not only reduce, but also sometimes augment vector competence – and hence possibly pathogen transmission – and emphasizes the importance of using natural host-pathogen associations, and not only laboratory models [32], [33], [80].

### Stage-, Sex-, Diet- and Tissue-specific Density of wFlu in Ae. Fluviatilis

**Figure 7:**
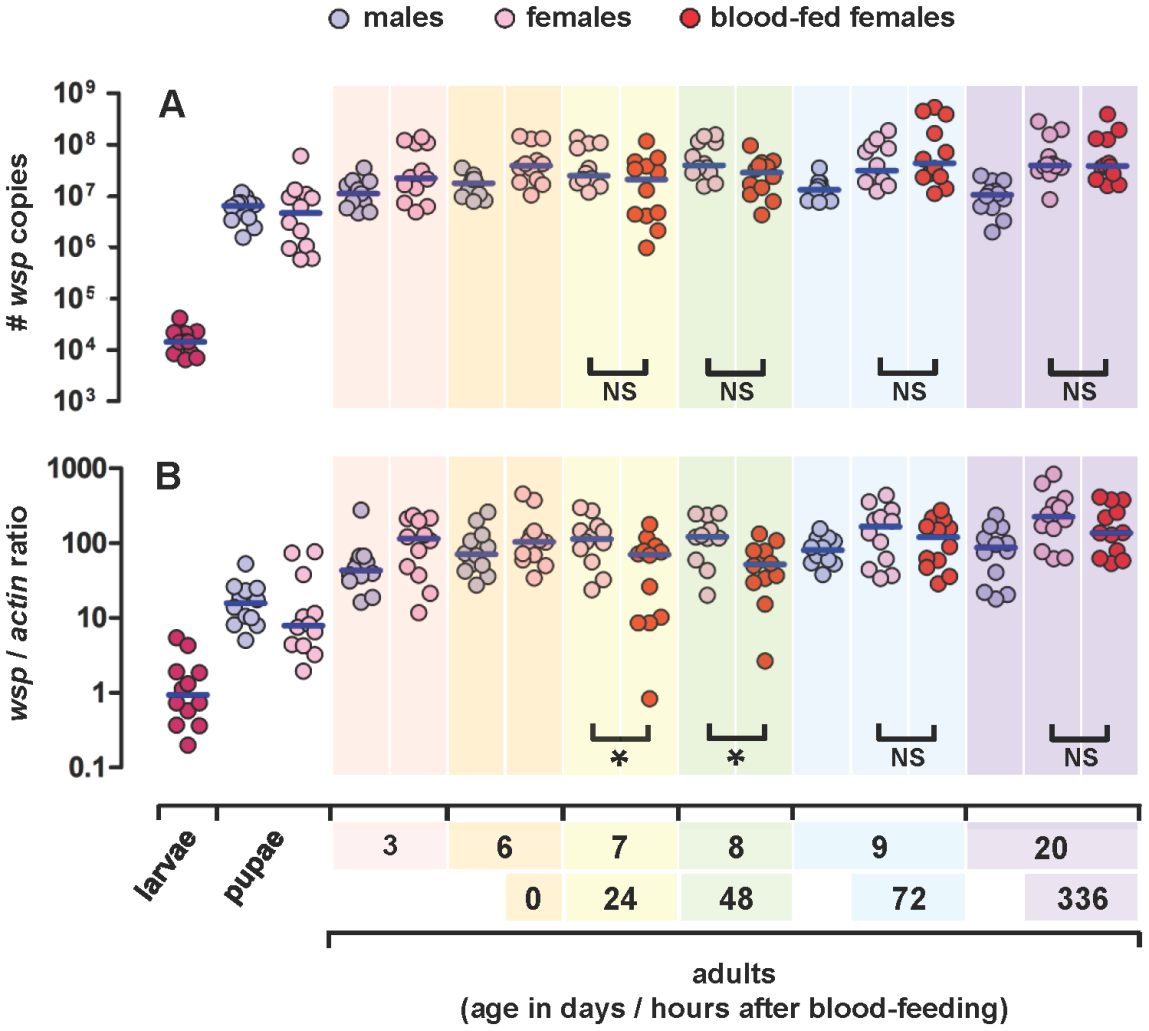
Stage-, sex-and diet-specific density of *w*Flu in *Ae. fluviatilis*. Graphs showing the absolute (A) and relative (B) densities of *w*Flu throughout the life cycle of the wildtype strain (*wolb^+^*) of the mosquito *Ae. fluviatilis*. The density of *w*Flu was estimated using real-time quantitative PCR of the *Wolbachia*-specific *wsp* gene and the mosquito-specific *actin* gene (see *Materials and Methods* for details). Each circle represents a single, whole individual, while the blue horizontal bars indicate either the median number of *wsp* copies (Graph A) or the median *wsp*/*actin* ratio (Graph B) per individual. The data shown are from three independent biological replicates (i.e., three different cohorts – generations – of the laboratory colony of *Ae. fluviatilis*). For each life cycle stage/sex/diet type, 4 individuals were assayed from each of the three cohorts, so that in total 12 individuals were used. For each cohort, adult females were separated into two groups 6 days after eclosion from pupae, and one group was blood-fed on the same day, such that 7, 8, 9 and 20 day-old adults are, respectively, 24, 48, 72 and 336 hours after blood-feeding, while the other group of age-matched adult females was maintained on sugar only. After day 9, blood-fed females were allowed to oviposit, so that fully-developed eggs would not be retained. As the sex of larvae cannot currently be unambiguously determined for aedine mosquitoes, only a single group representing an unknown mix of randomly selected male and female 4^th^ instar individuals was assayed. Comparisons marked with an asterisk (*) were significantly different between sugar- and blood-fed females using a Mann-Whitney *U* test, while comparisons marked with “NS” were not significantly different between sugar- and blood-fed females. Statistically significant differences were also observed between different life cycle stages and sexes as described in the main text.

**Figure 8:**
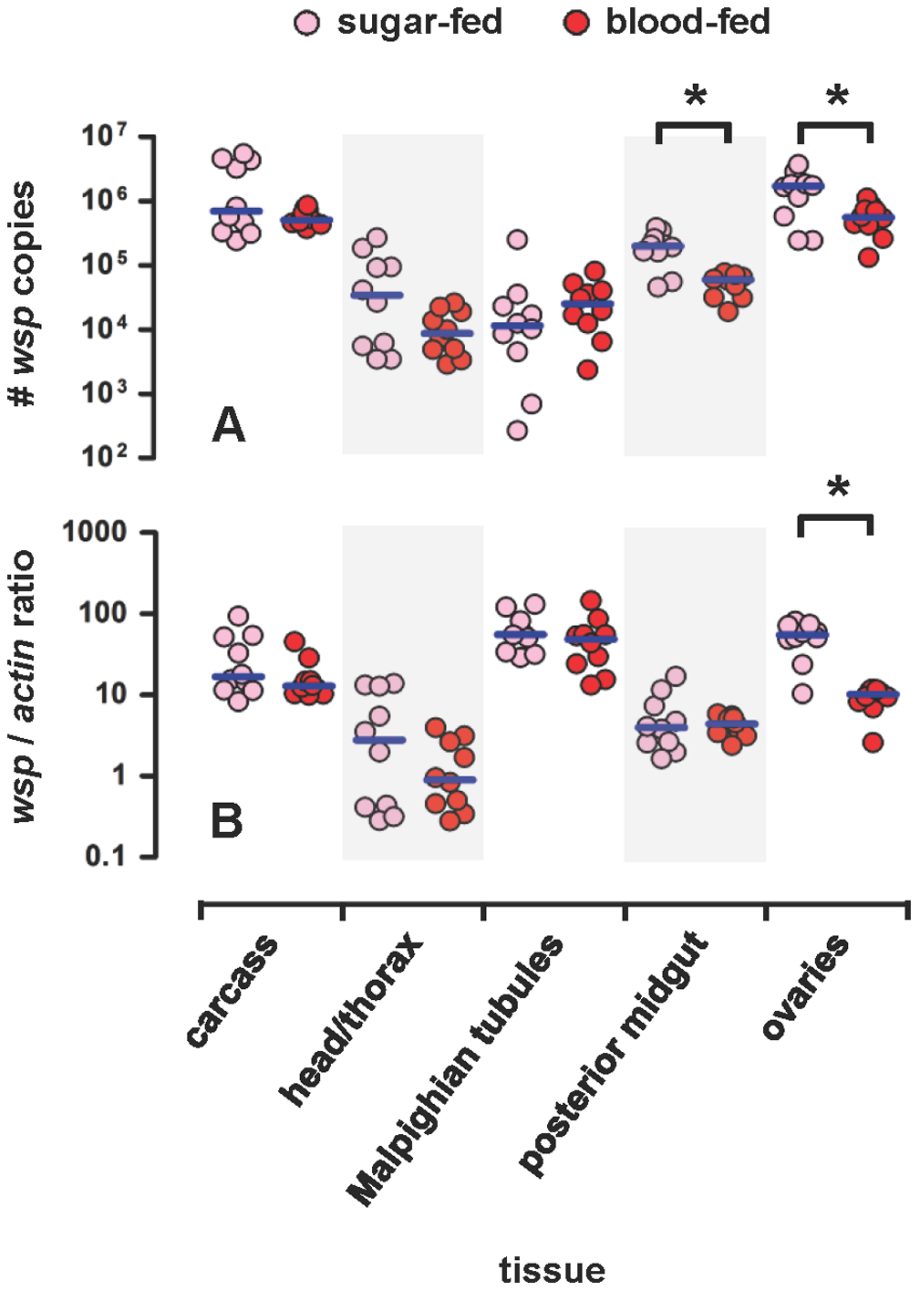
Tissue-specific density of *w*Flu in sugar- and blood-fed adult female *Ae. fluviatilis*. Graphs showing the absolute (A) and relative (B) densities of *w*Flu in different tissues of adult females of the wildtype strain (*wolb^+^*) of the mosquito *Ae. fluviatilis*. The density of *w*Flu was estimated using real-time quantitative PCR of the *Wolbachia*-specific *wsp* gene and the mosquito-specific *actin* gene (see *Materials and Methods* for details). Each circle represents a single pool of 5 individual organs taken from different age- and cohort-matched individuals, while the blue horizontal bars indicate either the median number of *wsp* copies (Graph A) or the median *wsp*/*actin* ratio (Graph B) per individual. The data shown are from two independent biological replicates (i.e., two different generations of the laboratory colony of *Ae. fluviatilis*). Three to 5 day-old adult females were separated into two groups after eclosion from pupae, and one group was blood-fed, while the other was maintained on sugar only. Twenty-four hours later (i.e., after blood-feeding, when the females were 4 to 6 days old), both sugar-fed and blood-fed individuals were dissected, and their organs harvested. In graph A, the absolute density of *w*Flu per *individual* organ was estimated by dividing the calculated number of *wsp* copies for each sample (i.e., pool of organs) by the number of organs in each pool (i.e., 5 organs). The cohorts (i.e., generations) of mosquitoes assayed were different from those used in Figure 7, such that the data presented in the two figures are not directly comparable, although they give consistent results. Comparisons marked with an asterisk (*) were significantly different between sugar- and blood-fed females using a Mann-Whitney *U* test, while unmarked comparisons were not significantly different between sugar- and blood-fed females. Statistically significant differences were also observed between some of the different tissues as described in the main text.

The density and tissue distribution of *Wolbachia* within its hosts is thought to determine a number of characteristics of the host-endosymbiont association [107], including: (i) the expression of cytoplasmic incompatibility (see discussion above), (ii) the virulence of *Wolbachia* to its host (i.e., the life-shortening and other pathological effects) [35], [36], [85], [97], and (iii) pathogen interference [2], [27], [108], [109]. Accordingly, in order to gain further insight into these traits, both the absolute and relative stage-, sex- and tissue-specific densities of *w*Flu in individuals of the untreated wildtype (*wolb^+^*) *Ae. fluviatilis* strain were determined using real-time quantitative PCR (Figures 7 and 8).

As expected from, and consistent with, previous light and electron microscopic studies [7], [8], [110]–[112], as well as other quantitative PCR investigations [113]–[117], using other mosquito species naturally-infected with different strains of *Wolbachia*, the density of *w*Flu varied across the life cycle of *Ae. fluviatilis*, being low in larvae and increasing dramatically in pupae, and then again in adults, of both sexes, especially in females (Figure 7). The absolute and relative densities of *w*Flu were significantly different between the three different life cycle stages (larvae/pupae/adults) assayed (Kruskal-Wallis test: absolute densities, *P*<0.0001; and relative densities *P*<0.0001). The absolute and relative densities of *w*Flu did not differ between male and female pupae (Mann-Whitney *U* test: absolute densities, *P* = 0.1939; and relative densities *P* = 0.6650). The absolute and relative densities of *w*Flu did not significantly change, within either sex, with adult age, for sugar-fed individuals (Kruskal-Wallis test: males, absolute densities *P* = 0.2189; and relative densities, *P* = 0.1134; and females, absolute densities *P* = 0.4561; and relative densities, *P* = 0.2028), but were both significantly higher in adult sugar-fed females than age-matched male adults (Mann-Whitney *U* test: absolute densities *P*<0.0001; and relative densities, *P* = 0.0004). Although there is considerable variation between individuals, *w*Flu densities in adult males, however, appeared to initially increase and then subsequently decline with increasing male age (Figure 7 and Supporting Information, Figure S1), suggesting that the ability of this *Wolbachia* strain to induce cytoplasmic incompatibility might vary in age-dependent manner, as reported for some mosquito-*Wolbachia* associations (see also discussion on cytoplasmic incompatibility above) [60], [91], [117]–[119]. The high heterogeneity observed in *w*Flu densities between individual adult females may also account for the observed variable effect of this *Wolbachia* strain on *P. gallinaceum* oocyst infection (see above and Figures 5 and 6).

Consistent with previous studies using indirect immunofluorescence (IFA) and fluorescent *in situ* hybridization (FISH) [27], *w*Flu was detected in the head/thorax, Malpighian tubules and ovaries (Figure 8). However, in contrast to previous work [27], considerable absolute levels of *w*Flu were also detected in the carcass and posterior midgut, although the relative levels were low (Figure 8). A similar discrepancy between the results of FISH and quantitative PCR detection of *Wolbachia* in mosquito midguts has been previously reported [120], and may reflect differences in the sensitivity of the two detection methods, or possible contamination of the midgut with *Wolbachia* from other tissues (especially any remnants of the Malpighian tubules, but also the trachea and musculature associated with the midgut, not removed from the midgut during dissection).

The absolute and relative densities of *w*Flu both differed significantly between the different tissues of sugar-fed adult females (Kruskal-Wallis test: absolute densities, *P*<0.0001; and relative densities *P*<0.0001) (Figure 8). The absolute and relative densities of *w*Flu exhibited similar patterns in the head/thorax, posterior midgut and ovaries of adult sugar-fed females (i.e., the two measures of density were either both low or both high for each tissue). However, in the other two tissues examined, contrasting density patterns were observed: in the carcass absolute densities were among the highest, while relative densities were low; and the converse pattern was observed in the Malpighian tubules (i.e., absolute densities were the lowest observed, while relative densities were among the highest) (Figure 8). These contrasting patterns of absolute and relative density of *w*Flu in the carcass and Malpighian tubules can be explained by differences in the relative sizes of these tissues: the former is very large, while the latter is comparatively very small. Consistent with previous microscopic studies [7], [8], [110]–[112], and the general biology of *Wolbachia* as a maternally-inherited symbiont that manipulates host reproduction [9], the highest densities of *w*Flu – both relative and absolute – were observed in the ovaries of *Ae. fluviatilis* (Figure 8).

We also determined the density of *w*Flu in whole adult females and their different tissues at various times after bloodfeeding (Figures 7 and 8). In contrast with sugar-fed adult females, the relative density of *w*Flu significantly changed with age in blood-fed adult females (Kruskal-Wallis test, *P* = 0.0051), while there was a similar, but marginally non-significant, trend with absolute densities (Kruskal-Wallis test, *P* = 0.0819) (Figure 7). Pairwise comparisons further showed that the relative density of *w*Flu was significantly lower at 24 and 48 hours, but not at 72 hours and 14 days, after bloodfeeding (days 7, 8, 9 and 20 respectively in Figure 7), than in age-matched sugar-fed females from the same cohorts of mosquitoes (Mann-Whitney *U* test: day 7, *P* = 0.0262; day 8, *P* = 0.0086; day 9, *P* = 0.4357; and day 20, *P* = 0.3123) (Figure 7). In contrast, the absolute density of *w*Flu was not significantly different at any time between age-matched sugar- and blood-fed adult females, although the observed values tended to be lower in blood-fed females at 24 and 48 hours (days 7 and 8, respectively), and were only marginally non-significant at the latter time (Mann-Whitney *U* test: day 7, *P* = 0.2145; day 8, *P* = 0.0783; day 9, *P* = 0.4357; and day 20, *P* = 0.7950) (Figure 7). Overall, these data seem to indicate that there is a reduction in *Wolbachia* densities in the 48 hour period after blood-feeding, after which the numbers of *w*Flu return to levels comparable to those observed in age-matched sugar-fed females. These observations contrast with those of artificial *w*MelPop infection of mosquitoes, where *Wolbachia* density increases following bloodfeeding and is associated with reduced host survival [30], [33].

The tissue-specific density of *w*Flu in adult females 24 hours after blood-feeding was similar to that observed in age-matched sugar-fed individuals, except for the ovaries (Figure 8). The absolute and relative tissue-specific densities of *w*Flu did not differ significantly between sugar- and blood-fed adult females for the carcass, head/thorax, posterior midgut and Malpighian tubules (Mann-Whitney *U* test: absolute densities, *P*>0.1051, in all instances, except for midguts, which were significantly lower in blood-fed females, *P* = 0.0039; and relative densities, *P*>0.2176 in all instances). In contrast, both the absolute and relative densities of *w*Flu were significantly lower in ovaries from blood-fed females (Mann-Whitney *U* test: absolute densities, *P* = 0.0355; and relative densities, *P* = 0.0002) (Figure 8). This reduction in the detection of *Wolbachia* in the ovaries of blood-fed females is consistent with the reduced density of *w*Flu observed in whole adult females at 24 and 48 hours after blood-feeding (days 7 and 8, respectively) (see above and Figure 7). The lower *absolute* density of *w*Flu observed in the ovaries, and possibly also in whole adult females, suggests that *Wolbachia* are lost during the period of egg development that follows blood-feeding (i.e., the relative density of *wsp* gene copies is not lower merely because of an increase in the number of mosquito host genomes following blood-feeding).

The significance of this surprising observation, that *w*Flu densities are lower in the ovaries of blood-fed females, is uncertain, and further work is required to confirm and understand what is happening to the density of *Wolbachia* during the resumption of oogenesis that follows bloodfeeding in mosquitoes. A reduction in *Wolbachia* densities following bloodfeeding has not previously been reported (although it is possibly suggested by Figure 3B in [96]). Previous studies have observed degenerate and pathological ovarian cells associated with natural *Wolbachia* infection of mosquitoes, as well as *Wolbachia* disintegration and absorption within ovaries [8], [110], [111], [121]–[125]. However, there are no systematic and quantitative studies that have determined whether the occurrence in mosquito ovaries of such degenerate *Wolbachia* and host cells are increased following bloodfeeding, and during the mid- and/or late stages of oogenesis. During oogenesis in the fruit fly *Drosophila*, *Wolbachia* along with other cytoplasmic contents are transferred from nurse cells through intercellular bridges to their associated oocyte by a process termed “cytoplasmic dumping”, after which the nurse cells undergo programmed cell death and removal from the developing egg [126], [127]. The equivalent processes in mosquitoes are not well characterized, and in at least some dipterans cytoplasmic dumping is known not to occur [128]. Interestingly, in *Ae. fluviatilis* the density of *w*Flu, as determined by FISH, is highest in ovarian nurse cells [27]. If cytoplasmic dumping, and hence the transfer of *Wolbachia* from nurse cells to the oocyte, does not occur in mosquitoes, then programmed cell death of nurse cells, which is a normal component of mosquito oogenesis [129], could account for the reduction of *w*Flu densities observed in the ovaries of blood-fed mosquitoes. Whatever the cause of the loss of *w*Flu from the ovaries following bloodfeeding, it does not seem to affect host fecundity, as equivalent numbers of eggs are laid by wildtype (*wolb^+^*) and antibiotic-treated (*wolb^−^*) strains of *Ae. fluviatilis* (see above and Figure 3) (i.e., the reduction in the density of *w*Flu apparently does not result from elevated levels of oocyte degeneration in *Wolbachia*-infected females).

### Conclusions

Overall, our observations indicate that the *Wolbachia* strain *w*Flu has the potential to be used as a vector control agent. *w*Flu causes high levels of cytoplasmic incompatibility, has efficient maternal transmission, and no apparent fitness costs, indicating that it will rapidly spread through host populations, and is seemingly suitable as a gene drive mechanism [17]–[20] or for direct suppression of host populations using release of incompatible males [15], [16], [19]. The apparent absence of virulence and pathogen interference/symbiont-meditated protection we observed with *w*Flu in its native host *Ae. fluviatilis* is consistent with its low density and limited tissue distribution, and is indicative of appreciable long-term mutualistic coevolution between this host and its endosymbiont [73], [84]. These observations suggest that *w*Flu will have only a limited, if any, ability to modify the age-structure of vector populations, and hence pathogen transmission, through reducing vector longevity [37]–[40]. However, further future research will be required to determine whether *w*Flu has similar or different effects when transferred to the novel mosquito hosts that are the vectors of human pathogens. Current research suggests that it is unlikely that *w*Flu will reduce vector survival (i.e., be virulent in a new host), as the life-shortening and other density-related virulence effects [35], [36] of *Wolbachia* appear to be strain-specific, rather than determined by host background [25], [31], [85]. In contrast, avirulent native *Wolbachia* can induce pathogen interference when transferred to novel artificial hosts [28], [30], [98], and have opposing effects on different parasite species [33], providing hope that *w*Flu may also directly inhibit human pathogens when artificially-transfected into their natural mosquito vectors.

## Supporting Information

Figure S1 **Age-related changes in the density of** ***w*** **Flu in male** ***Ae. fluviatilis*** **.** Graphs showing the absolute (A) and relative (B) densities of *w*Flu in males of the wildtype strain (*wolb* ^+^) of the mosquito *Ae. fluviatilis*.
(PDF)
