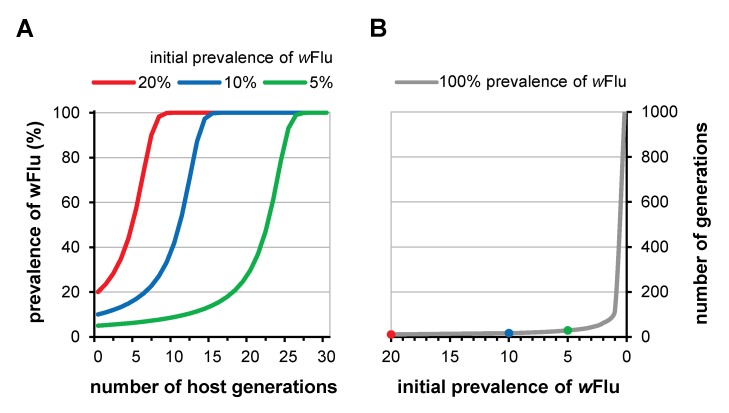# Correction: *w*Flu: Characterization and Evaluation of a Native *Wolbachia* from the Mosquito *Aedes fluviatilis* as a Potential Vector Control Agent

**DOI:** 10.1371/annotation/310194a4-e502-4693-a01a-309e4ebdb64b

**Published:** 2013-10-24

**Authors:** Luke Anthony Baton, Etiene Casagrande Pacidônio, Daniela da Silva Gonçalves, Luciano Andrade Moreira

In Figure 4, three colored circles are missing from the line of Graph B. Please see the corrected Figure 4 here: 

**Figure:**