# Supplementary material for: wFlu: Characterization and Evaluation of a Native Wolbachia from the Mosquito Aedes fluviatilis as a Potential Vector Control Agent
Source: PLoS One. 2013 Mar 26;8(3):e59619. doi: 10.1371/journal.pone.0059619 (PMC3608659; doi:10.1371/journal.pone.0059619)
Supplement: Figure S1 — Age-related changes in the density of w Flu in male Ae. fluviatilis . Graphs showing the absolute (A) and relative (B) densities of wFlu in males of the wildtype strain (wolb +) of the mosquito Ae. fluviatilis. (PDF) [file pone.0059619.s001.pdf]

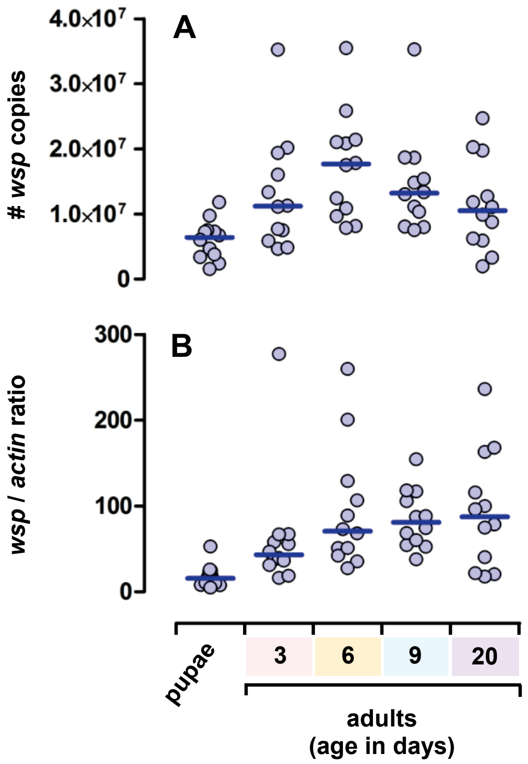

Figure S1. Age-related changes in the density of *w*Flu in male *Ae. fluviatilis*.

Graphs showing the absolute (A) and relative (B) densities of *w*Flu in males of the wildtype strain (*wolb*$^{+}$) of the mosquito *Ae. fluviatilis*. Data shown are the same as those presented in Figure 7 of the main manuscript, but shown using a linear, rather than logarithmic, scale (and excluding the data for larvae and females). The density of *w*Flu was estimated using real-time quantitative PCR of the *Wolbachia*-specific *wsp* gene and the mosquito-specific *actin* gene (see *Materials and Methods* for details). Each circle represents a single, whole individual, while the blue horizontal bars indicate either the median number of *wsp* copies (Graph A) or the median *wsp*/*actin* ratio (Graph B) per individual. Note that the absolute and relative densities of *w*Flu correspond in pupae and young adult males, but exhibit contrasting patterns in older adult males. Although the absolute and relative densities of *w*Flu did not significantly change with adult age (Kruskal-Wallis test: males, absolute densities $P = 0.2189$; and relative densities, $P = 0.1134$), the observed data suggest that the absolute densities of *w*Flu initially increase

and then decline with increasing adult age, while the relative densities either increase, or increase and then plateau, with increasing adult age. The apparent decline in absolute densities of *w*Flu in older adult males, without a corresponding decrease in the relative density of *w*Flu, implies, and would be consistent with, a decline in host cell numbers with increasing age. This failure to detect a significant relationship between the density of *w*Flu and adult age probably reflects (i) the relatively small sample sizes used ($n = 12$ for each life cycle stage/age), (ii) and the existence of high heterogeneity in the density of *w*Flu between individuals (both within and between colony generations; unpublished observations).

Our observations indicate the importance of determining both absolute and relative densities of *Wolbachia*. For example, Duron et al [1] reported that densities of the *Wolbachia* strain *w*Pip were higher in the testes of 30 than 2-day-old *Culex pipiens* mosquitoes, but these authors only reported relative densities. Our observations suggest that the relative densities of *w*Flu in adult male *Ae. fluviatilis* exhibit a similar pattern to those of *w*Pip in the testes of *Cx. pipiens*, but also indicate that this increase is not due to a corresponding increase in the absolute densities of *Wolbachia*.

References

1. Duron O, Fort P, Weill M (2007) Influence of aging on cytoplasmic incompatibility, sperm modification and *Wolbachia* density in *Culex pipiens* mosquitoes. Heredity (Edinb) 98: 368-374. 10.1038/sj.hdy.6800948.
